# Case Report: long-term clinical outcomes in *RANBP2*-associated acute necrotizing encephalopathy

**DOI:** 10.3389/fphar.2025.1607682

**Published:** 2025-06-05

**Authors:** Sonia A. Varghese, Olutayo I. Olubiyi, Irena Dujmovic Basuroski, Jordan Broman-Fulks, Emma B. Cardwell, Stephanie Peck, Qian-Zhou (JoJo) Yang, Sheng-Che Hung, Senyene E. Hunter

**Affiliations:** ^1^ Department of Pediatrics, Wilmington Pediatric Specialty Division, University of North Carolina, Wilmington, NC, United States; ^2^ Commonwealth Radiology, Richmond, VA, United States; ^3^ Department of Neurology, University of North Carolina, Chapel Hill, NC, United States; ^4^ Department of Radiology, University of North Carolina, Chapel Hill, NC, United States; ^5^ Biomedical Research Imaging Center, University of North Carolina, Chapel Hill, NC, United States

**Keywords:** acute necrotizing encephalopathy, RANBP2 variant, brain MRI, viral infection, immunomodulation, case report

## Abstract

**Introduction:**

Acute necrotizing encephalopathy (ANE) is a rare and severe neurological condition primarily affecting children and commonly triggered by viral infections. Morbidity and mortality rates are high. Pathogenic RAN-Binding Protein-2 (*RANBP2*) variants predispose children to recurrent ANE, known as ANE1, and increase the risk of severe outcomes and early death. Although the pathophysiology of ANE is not fully understood, an inflammation-mediated “cytokine storm” is believed to play a crucial role in central nervous system involvement. Currently, there is no guidance on the optimal duration of immunotherapy.

**Case presentation:**

We present a new pediatric case of *RANBP2*-associated ANE1, and update one previously published case, detailing their clinical characteristics, treatment strategies, and outcomes. Magnetic resonance imaging revealed lesions characteristic of ANE. In one patient, cerebrospinal fluid (CSF) analysis showed pleocytosis without evidence of bacterial or viral pathogens, and elevated CSF levels of interleukin-6 (IL-6) and IL-8 were consistent with neuroinflammatory response. Both patients experienced rapid neurological decline during ANE attacks. However, both patients were treated with timely immunotherapy, including steroids, plasma exchange, intravenous immunoglobulins, and tocilizumab, with favorable responses.

**Conclusion:**

Recurrent ANE or ANE with a family history of severe neurological events in childhood should raise suspicion for *RANBP2*-associated ANE1. These cases emphasize the importance of early recognition, prompt immunotherapy initiation, and close monitoring in patients with ANE1. Our cases also contribute to the limited body of knowledge on neuroimaging, treatment, and outcomes in this rare condition, which is of great importance given that the optimal duration of immunotherapy in ANE1 is currently unknown.

## 1 Introduction

Acute necrotizing encephalopathy (ANE) is a rare, severe neurological condition characterized by rapid and potentially devastating decline, often triggered by infections such as influenza or SARS-CoV2 (Poyiadji et al., 2020; Wong et al., 2006; Wu et al., 2015). ANE presents with rapid-onset encephalopathy, often accompanied by seizures, focal deficits, and fever without evidence of infectious encephalitis (Lee et al., 2014; Bashiri et al., 2020; Neilson et al., 2009; Christou et al., 2024). Diagnostic criteria outlined by Mazuguchi (1997), includes: acute encephalopathy after febrile illness; elevated cerebrospinal fluid (CSF) protein without pleocytosis; symmetric multifocal lesions involving the bilateral thalami, periventricular white matter, internal capsule, putamen, upper brainstem, and cerebellar medulla; and elevated serum aminotransferase (Mizuguchi, 1997; Ichiyama et al., 2003). Systemic symptoms, including multiorgan failure and systemic inflammatory response syndrome, may also occur. Outcomes range from complete recovery to death (Levine et al., 2020; Mizuguchi et al., 1995), although the factors contributing to these varied outcomes remain unclear.

The pathophysiology of ANE is not fully understood, but emerging evidence suggests that dysregulated cytokine production plays a major role. The hypothesis of a cytokine-mediated mechanism is supported by findings of elevated cytokine levels, such as interleukin 6 (IL-6) and tumor necrosis factor-alpha (TNF-alpha), in the serum and CSF of affected individuals (Shukla et al., 2021). Treatment typically involves prompt initiation of immunotherapy, which is thought to mitigate the inflammation-mediated “cytokine storm” underlying central nervous system (CNS) involvement (Shukla et al., 2021; Brattsand and Linden, 1996). Commonly used therapies include steroids, intravenous immunoglobulin (IVIG), plasma exchange (PLEX, replaces plasma with colloid fluid to reduce circulating inflammatory mediators), and tocilizumab. However, a standardized treatment protocol for recurrent ANE is lacking.

Familial forms of ANE have been associated with several genetic variants, most notably the nuclear pore protein RAN-Binding Protein 2 (*RANBP2*) (Neilson et al., 2009; Singh et al., 2015; Sondhi et al., 2016). Pathogenic *RANBP2* variants predispose individuals to recurrent ANE episodes, each of which increases the risk of severe neurological impairment or early death. This subset of familial ANE cases has been termed ANE1 (Singh et al., 2015), distinguishing this condition from sporadic cases (Neilson et al., 2009). Despite advancements in understanding the genetic underpinnings of ANE1, the condition remains challenging to diagnose and manage.

Currently, there is no guidance on the optimal duration of immunotherapy for ANE1. Here, we present a new pediatric case of *RANBP2*-associated ANE1 and provide updates on the treatment and resulting outcomes of a previously reported case (Olubiyi et al., 2022). Additionally, we review key neuroimaging and neuroimmunology findings in ANE1, adding to the sparse literature on this condition. Both patients experienced rapid neurological decline during the ANE1 episodes but had favorable responses following timely immunotherapy. These cases underscore the importance of early recognition and prompt immunotherapy initiation to optimize outcomes and contribute to the limited body of knowledge on outcomes in this rare condition, which is of great importance given that the optimal duration of immunotherapy in ANE1 is currently unknown.

## 2 Case description

### 2.1 Case 1

**FIGURE 1:**
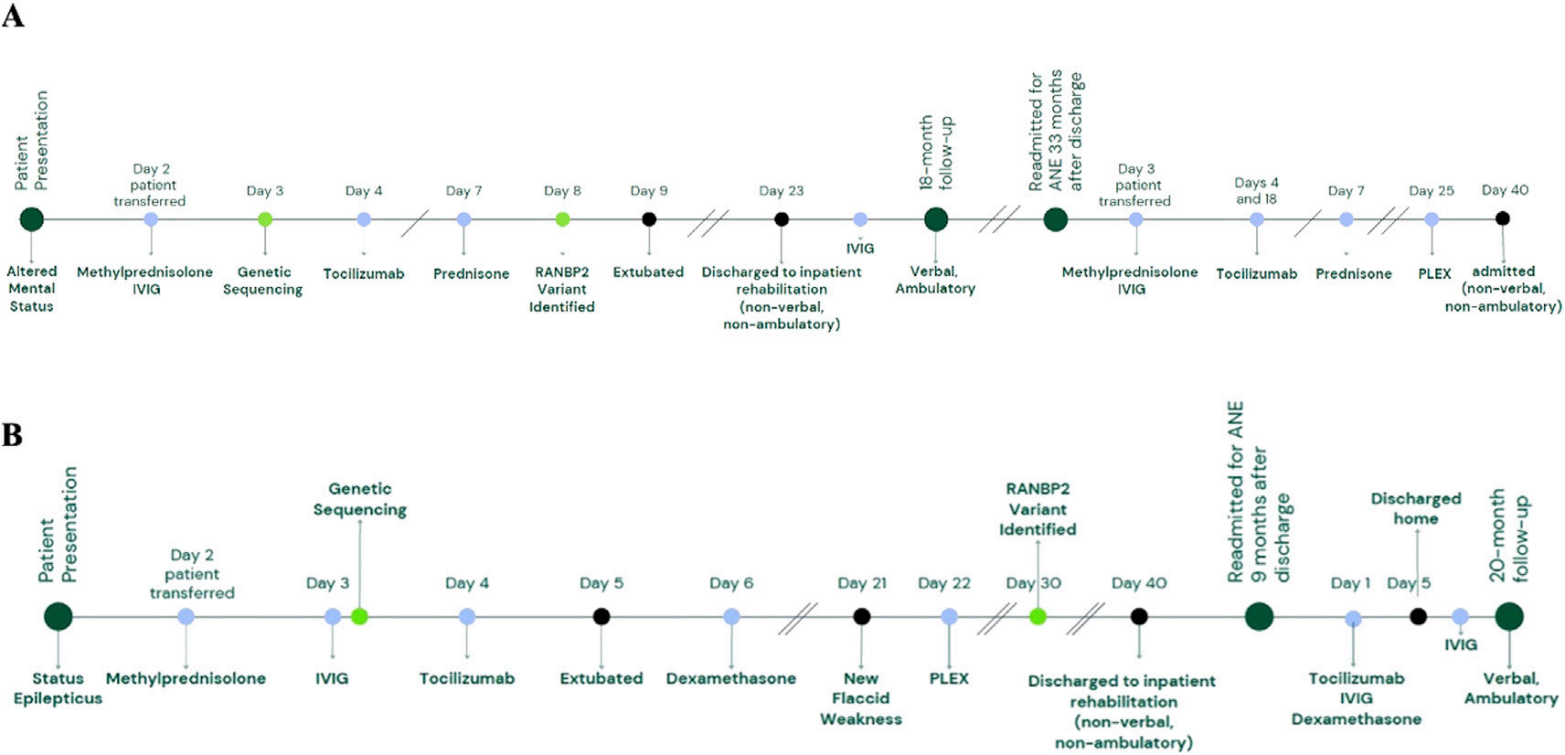
Timelines of clinical events. Timelines illustrate key clinical milestones during and after the first and second admissions for Case 1 at ages 23 and 58 months (4 years, 10 months) **(A)** and the second and third admissions for Case 2 at ages 30 and 40 months, respectively **(B)**. Clinical events are marked with black circles, immunotherapies with blue circles, and genetic sequencing with green circles.

**FIGURE 2:**
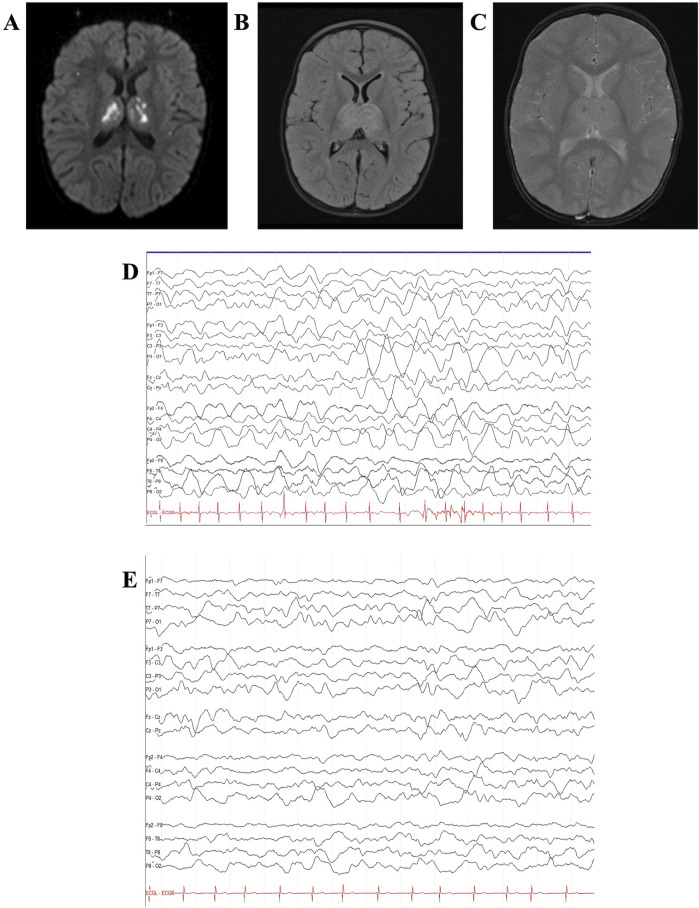
Case 1 MRI and EEG. Case 1 brain MRI at age 23 months demonstrated multiple foci of diffusion restriction on diffusion-weighted imaging **(A)** with associated hyperintensity on fluid-attenuation inversion recovery (FLAIR) images **(B)** and microhemorrhages on gradient-echo images **(C)** in bilateral thalami, prompting concern for acute necrotizing encephalopathy. Continuous video EEG (bipolar montage, sensitivity 15 uV/mm, timebase 30 mm/s) initially showed severe diffuse slowing with delta waveforms. **(D)** With improvement 1 day after tocilizumab administration **(E)**.

A previously healthy 23-month-old female presented with lethargy and acute-onset encephalopathy following a 2-day history of fever (Figure 1A). On neurological exam, she was responsive to tactile stimuli and had intermittent extensor posturing. A respiratory pathogen panel was positive for human rhinovirus/enterovirus and parainfluenza. CSF analysis revealed an elevated protein level (64 mg/dL), as shown in Table 1, no CSF pleocytosis, and negative CSF cultures and viral pathogen panels. Brain magnetic resonance imaging (MRI) was consistent with ANE, including bilateral thalamic T2/FLAIR hyperintensities (shown in Figures 2A–C). She was transferred to our facility on hospital day 2.

**TABLE 1:** Serum and CSF studies in two cases of ANE1.

	Laboratory test (unit)	Case 1		Case 2			References range
Age	(months)	23	58	25	30	40	
Blood studies	WBC (10^9^/L)	**2.5**	**1.8**	**3.0**	**17.9**	7.3	5.3–13.2
	Platelets (10^9^/L)	**99**	**170**	284	307	336	212–480
	AST (U/L)	43	**83**	33	54	27	<34
	ALT (U/L)	18	**80**	10	12	9	10–49
	LDH (U/L)	**271**	-	703	666	-	500–920
	CK (U/L)	70	-	-	-	-	34–145
	Albumin (g/dL)	**3.3**	**3.0**	**3.3**	**3.4**	3.9	3.5–5.0
	D-Dimer (ng/mL)	**598**	**4,755**	**263**	-	-	<230
	Pro-BNP (pg/mL)	-	-	**654**	-	-	0.0–93.0
	Fibrinogen (mg/dL)	336	380	304	**394**	-	177–386
	CRP (mg/L)	<4.0	**10**	<5.0	<5.0	<4.0	≤ 10.0
	ESR (mm/h)	**18**	**44**	**15**	-	**19**	0–13
CSF studies	CSF/serum albumin quotient	-	-	-	**26.6**	-	^*^0.5–4.0
	RBC (/mm^3^)	3	-	2	5	-	0
	WBC (/mm^3^)	1	-	**16**	**43**	-	^**^0–20
	CSF/serum glucose quotient	0.86	-	0.61	0.59	-	≥0.5
	Protein (mg/dL)	**64**	**-**	**85**	**182**	-	15–45
	CSF oligoclonal IgG bands	Negative	-	-	-	-	Negative
	IgG (mg/dL)	-	-	-	**10.6**	-	<7.0
	IL-1beta (pg/mL)	-	-	-	<5	-	≤5
	IL-2 (pg/mL)	-	-	-	**3**	-	≤1
	IL-4 (pg/mL)	-	-	-	**44**	-	≤7
	IL-5 (pg/mL)	-	-	-	2	-	≤2
	IL-6 (pg/mL)	-	-	-	**1,596**	-	≤25
	IL-8 (pg/mL)	-	-	-	**3,225**	-	≤205
	IL-10 (pg/mL)	-	-	-	**8**	-	≤2
	IFN gamma (pg/mL)	-	-	-	2	-	≤4
	TNF alpha (pg/mL)	-	-	-	1	-	≤4
	GM-CSF (pg/mL)	-	-	-	**3**	-	≤1

Abnormal findings are in bold. Hyphen (-) indicates that the study was not obtained. *Reference range for age 4 months-5 years. **Reference range for age 1-4 years. Abbreviations: RBC: Red blood cells; WBC: White blood cells; IgG: Immunoglobulin G; IL: Interleukin; IFN: Interferon; TNF: Tumor Necrosis Factor; GM-CSF: Granulocyte-Macrophage Colony Stimulating Factor; AST: Aspartate transaminase, ALT: Alanine aminotransferase, LDH: Lactase dehydrogenase, CK: Creatine kinase, CRP: C-reactive protein, ESR: Erythrocyte sedimentation rate, CSF: Cerebral spinal fluid, BNP: Brain natriuretic peptide..

Upon transfer, her ANE severity score (Yamamoto et al., 2015) was seven, indicating a high risk of severe neurological sequelae or death. Treatment initiated on admission included IV methylprednisolone 30 mg/kg/day and IVIG 2 g/kg divided over 2 days. Electroencephalogram (EEG) revealed severe diffuse slowing with predominant delta waveforms (shown in Figures 2D,E). Two days after transfer, tocilizumab 12 mg/kg was administered, resulting in mild interval improvement on EEG, with persistent severe diffuse slowing but addition of faster theta frequencies and improvement in the neurologic exam the following day (spontaneous cough and gag and eye-opening to stimulation). Methylprednisolone 30 mg/kg/day was administered for 5 days, followed by a transition to prednisolone 1 mg/kg/day for 1 month, with a subsequent gradual taper over 4 months.

Family history revealed two maternal uncles with similar presentations of acute encephalopathy at ages 16 and 22 months, both deceased, with working diagnoses of Leigh-like disease. Given this history, rapid trio whole exome sequencing (GeneDx Test #J774) was performed during admission, revealing a maternally inherited pathogenic *RANBP2* c.1754C>G (p.Thr585Met) variant. Her father tested negative for the variant. Given the genetic susceptibility to recurrent ANE, tocilizumab 12 mg/kg infusions were continued every 2 weeks for 5 months, and she was maintained on monthly IVIG 1 mg/kg infusions. She was discharged to acute inpatient rehabilitation 23 days after her admission for management of expressive speech (non-verbal) and motor delays (including inability to stand independently). At discharge, her Modified Rankin Scale score (Banks and Marotta, 2007; Bigi et al., 2011) was five, indicating severe neurological disability.

#### 2.1.1 Case 1 follow-up

She showed significant improvement. At her 18-month follow-up, her Modified Rankin Scale score (Banks and Marotta, 2007; Bigi et al., 2011) was two, indicating slight disability. By age 3.5 years, she spoke in sentences with decreased fluency and impaired articulation, ambulated independently but with an occasional toe-walking gait, and her family noted considerable progress in motor functioning. She continued monthly IVIG 1 g/kg.

At age 4 years, 10 months, she presented with acute-onset encephalopathy (agitation, malaise) following 4 days of fever, dysphagia, and abdominal pain. She was transferred to our facility after 1 day of delirium (non-sensical speech) and minimal response to noxious stimuli on exam. ANE severity score was 4 (CSF not obtained), suggesting moderate risk of severe neurological sequelae or death. Respiratory pathogen panel was unremarkable. Brain MRI demonstrated multifocal T2/FLAIR hyperintense lesions consistent with ANE (shown in Supplementary Figure S1) without evidence of hemorrhage. Video EEG showed no seizures. Serum IL-6 was elevated (8.6, reference <6.4 pg/mL). Treatment included IVIG 2 mg/kg and IV methylprednisolone (30 mg/kg/day for 5 days), followed by oral prednisolone 2 mg/kg/day. Tocilizumab 12 mg/kg was initiated 1 day after hospital transfer and was associated with subsequent improvement in mental status (spontaneous eye opening, visual tracking). PLEX was initiated 25 days after admission, given insufficient recovery. Tocilizumab was discontinued after an incidental cecal pneumatosis was identified (without evidence of perforation or associated clinical symptoms). A steroid taper was continued, with plans to resume monthly IVIG. She currently remains admitted, is non-verbal, and non-ambulatory.

### 2.2 Case 2

**FIGURE 3:**
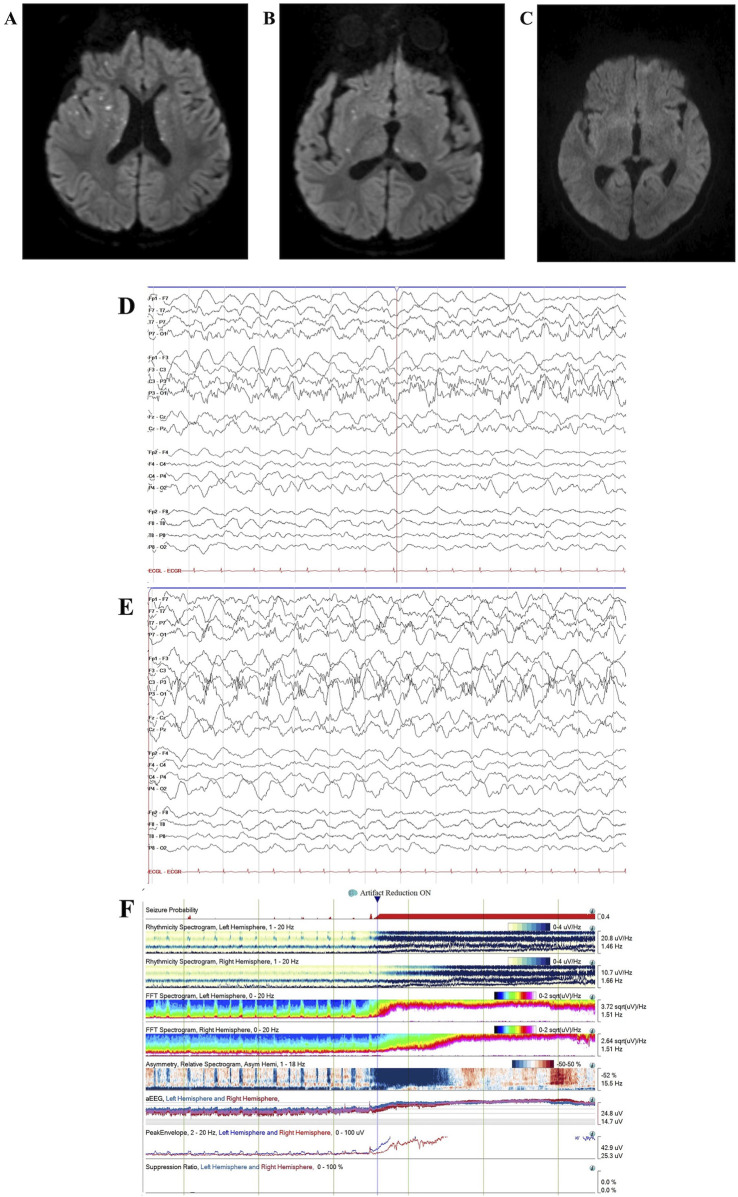
Case 2 MRI and EEG. Case 2 brain MRI age 40 months showed recurrent ANE with multiple new foci of diffusion restriction within bilateral basal ganglia, external and internal capsules, thalami, and corpus callosum on diffusion-weighted imaging, consistent with recurrent acute necrotizing encephalopathy **(A,B)**. Follow-up brain MRI at age 4.5 years showed interval resolution of basal ganglia/thalamic abnormalities with persistent pontine encephalomalacia **(C)**. Continuous video EEG (bipolar montage, sensitivity 15 uV/mm, timebase 30 mm/s) showed left hemisphere-onset seizures **(D)** with status epilepticus **(E)**. Quantitative EEG spectrogram and amplitude-integrated trends (2-h duration) showed frequent left hemisphere-onset seizures progressing to subclinical left hemisphere-onset status epilepticus with secondary generalization **(F)**.

A 24-month-old male, previously reported (Olubiyi et al., 2022), presented with acute encephalopathy, seizures, and emesis in the setting of a febrile illness (Figure 1B). Respiratory SARS-CoV-2 RNA PCR was positive. Brain MRI was concerning for ANE, including lesions in the thalamus as shown in Figures 3A–C (Olubiyi et al., 2022). He received IVIG 2 g/kg and dexamethasone 1 mg/kg daily (rapid taper finished by discharge), with clinical improvement to baseline on hospital day 4 and discharged home on day 7.

He again presented at age 30 months with acute-onset encephalopathy, delirium (including concern for visual hallucinations), and seizures (rhythmic eye jerking and apnea) following a 7-day prodrome of fever, emesis, and abdominal pain. As previously detailed (Olubiyi et al., 2022), CSF analysis revealed elevated protein (182 mg/dL), IL-6 (1,596 pg/mL), and IL-8 (3,225 pg/mL) (shown in Table 1). CSF cultures and viral pathogen panels were negative. Brain MRI was consistent with ANE recurrence, demonstrating increased T2/FLAIR hypertensities with associated restricted diffusion, along with mild enhancement within the obstructing bilateral midbrain and pons lesions (shown in Supplementary Figure S2) (Olubiyi et al., 2022).

This report provides additional details and updates the previously published case (Olubiyi et al., 2022). On hospital day one, the patient was transferred to our facility to address seizures and abnormal examination findings, including pupillary asymmetry and extremity posturing. His ANE severity score (Yamamoto et al., 2015) was six, indicating a high risk of severe neurological sequelae or death. Treatment on admission included IVIG 2 g/kg and a 5-day course of IV methylprednisolone 30 mg/kg/day, followed by dexamethasone 0.5 mg/kg/day with a gradual taper. EEG revealed subclinical focal-onset status epilepticus, with onset from the left posterior region (shown in Figures 3D,E), managed with fosphenytoin 30 mg/kg total, valproic acid 40 mg/kg, and maintenance levetiracetam 60 mg/kg/day. Two days after transfer, tocilizumab 12 mg/kg was administered, leading to mild improvement the following day, with spontaneous eye-opening and purposeful extremity movement. However, his neurological status subsequently worsened. He required PLEX 20 days after transfer for new flaccid weakness and progression of radiological findings on MRI, including marked petechial hemorrhage (Olubiyi et al., 2022). Following PLEX, he regained spontaneous antigravity movements.

Given his recurrent ANE, a commercial gene panel (Invitae Epilepsy Panel #03401) was obtained, revealing a maternally inherited likely pathogenic *RANBP2* c.1966A>G (p.Ile656Val) variant. His father tested negative for the variant. He was discharged 40 days post-transfer to acute inpatient rehabilitation for management of expressive speech disorder (non-verbal) and motor delays (inability to sit or stand independently). At hospital discharge, his Modified Rankin Scale (Banks and Marotta, 2007; Bigi et al., 2011) score was six, indicating risk of severe disability. Tocilizumab infusions (12 mg/kg every 2 weeks for 6 months) were continued during inpatient rehabilitation. Due to limited guidelines for long-term ANE1 management, parents declined maintenance IVIG and evaluation for inflammatory markers at that time.

He was readmitted at age 40 months for decreased attentiveness due to ANE (illustrated in Figure 1B) with associated bilateral thalamic restricted diffusion and FLAIR hyperintensities on MRI (shown in Supplementary Figure S3). Treatment upon readmission included tocilizumab 12 mg/kg, a 5-day course of dexamethasone 1 mg/kg/day with a subsequent taper, and IVIG 2 mg/kg followed by monthly IVIG 1 mg/kg infusions. His mental status improved 3 days after admission, and he returned to baseline by discharge on day 5. At discharge, his Modified Rankin Scale (Banks and Marotta, 2007; Bigi et al., 2011) score was three, indicating slight disability.

#### 2.2.1 Case 2 follow-up

He showed significant improvement. On neurological exam 14 months after discharge (age 4.5 years), he was forming sentences and ambulated independently with a mildly ataxic gait. Repeat MRI showed interval resolution of basal ganglia/thalamic abnormalities (shown in Figure 3C). Neuropsychological testing 20 months after discharge revealed cognitive impairment (WPPSI-IV IQ 65) and a mild mixed receptive-expressive language disorder, with overall age-appropriate functioning for a 5-year-old. His family reported near-baseline motor function, normal bilingual speech, and independent stair climbing. The patient continues treatment with monthly IVIG 1 g/kg.

## 3 Discussion

### 3.1 ANE and outcomes

We present a new pediatric case of *RANBP2*-associated ANE with two ANE episodes and updates on a previously reported case (Olubiyi et al., 2022) with three episodes. Both cases showed rapid neurological decline but with positive responses to treatment.

ANE is a rare, devastating condition typically triggered by viral infections, leading to rapid neurological deterioration. Although initially associated with a high mortality rate of 65% and severe neurological sequelae, recent studies reveal a broader spectrum of outcomes, from full recovery (<10%) to persistent deficits or death, with an estimated 30% mortality rate (Mizuguchi et al., 1995; Kim et al., 2004). The significant recovery in Case 1 (following her first ANE episode) and Case 2 (after recurrent ANE episodes) underscore the potential benefits of early, aggressive immunotherapy.

Several prognostic factors have been associated with poor outcomes in ANE, including age under 1 year, delirium associated with brainstem involvement, hemorrhagic lesions, brain tissue loss, high serum aminotransferases, CSF protein elevation, and thrombocytopenia (Levine et al., 2020; Okumura et al., 2009a). Higher ANE severity scores also correlate with poor outcomes (Yamamoto et al., 2015), while better outcomes are associated with unilateral thalamic lesions and lesion resolution (Levine et al., 2020).

Both cases exhibited multiple poor prognostic indicators typically associated with worse outcomes in ANE. For Case 1, poor prognostic features during the first ANE episode included bilateral thalamic and brainstem lesions with hemorrhage, mild CSF protein elevation, thrombocytopenia, and delirium. CSF cell count was normal. For Case 2, poor prognostic features included bilateral thalamic and brainstem lesions with hemorrhage, elevated CSF protein, CSF pleocytosis (a rare finding in ANE) (Shukla et al., 2021), and delirium. Early high-dose steroid administration is strongly linked to improved outcomes (Okumura et al., 2009b). Despite the presence of multiple high-risk features in both cases, early immunotherapy likely contributed to their favorable outcomes, particularly given the genetic underpinnings complicating their prognosis.

### 3.2 Neuroimaging features of ANE

Hallmarks of ANE observed in these cases include symmetric, multifocal necrosis affecting gray and white matter, primarily involving the thalami, tegmentum, cerebral white matter, internal capsule, putamen, and cerebellum (Olubiyi et al., 2022; Singh et al., 2015). While these patterns are well-described in ANE, neuroimaging features specific to ANE1 remain poorly defined due to its rarity, though recurrent cases often show lesions at varying stages and brain volume loss (Wong et al., 2006; Gika et al., 2010). As in the presented cases (shown in Supplementary Figures S1, S2), thalamic involvement in *RANBP2*-associated ANE is typically multifocal and punctate, which differs from the classic pattern seen in ANE (Singh et al., 2015). It is noteworthy that both patients demonstrated brainstem involvement, which, although not uncommon, has been associated with increased disease severity and poor outcomes, including higher mortality and long-term morbidity (Lee et al., 2014).

MRI predictors of ANE outcomes vary, with hemorrhage, cavitation, and lesion extent linked to worse prognosis (Wong et al., 2006), and brainstem lesions predictors of higher ANE severity scores (Yamamoto et al., 2015). In the current cases, brainstem involvement contributed to higher ANE severity scores of 7 and 6, indicating a significant risk of severe sequelae or death. Notably, Case 2 demonstrated hemorrhagic lesions, though not extensive, which may have contributed to his favorable outcome. Similarly, in a study comparing those with and without *RANBP2* variants, *RANBP2* was linked to a higher relapse risk but did not correlate with hemorrhagic lesions or prognosis (Chatur et al., 2022). The positive clinical and radiological recoveries of the presented cases highlight the importance of early, individualized interventions in improving outcomes. While our cases contribute to the limited knowledge, further research is needed to clarify the prognostic value of specific neuroimaging features in ANE1.

### 3.3 ANE1 pathogenesis

Understanding genetic influences in ANE, particularly *RANBP2*, is essential for clarifying outcome variability. Pathogenic *RANBP2* variants, inherited in an autosomal dominant fashion with 40% penetrance, predispose individuals to recurrent ANE, known as ANE1, with increased neurological risk (Neilson et al., 2009; Singh et al., 2015; Shen et al., 2021a; Christou et al., 2024). Our cases identified *RANBP2* p.Thr585Met and p.Ile656Val variants, both previously associated with ANE1, though specific genotype-phenotype correlations within ANE1 remain unclear (Neilson et al., 2009; Gika et al., 2010; Bergamino et al., 2012; Gilson et al., 2011; Hartley et al., 2021). The p.Thr585Met and p.Ile656Val variants are located in the N-terminal zinc finger region of RanBP2, and functional studies demonstrate that these variants disrupt factors that mediate suppression of IL-6 protein production (Shen et al., 2021b). The p.Thr585Met variant is classified in ClinVar as pathogenic/likely pathogenic based on American College of Medical Genetics (ACMG) guidelines (Richards et al., 2015; Landrum et al., 2018). Although ClinVar has conflicting pathogenicity classifications for the p.Ile656Val variant, tools using ACMG guidelines to determine pathogenicity predict that it is likely pathogenic (Richards et al., 2015). These cases highlight the role of genetic testing in ANE1 diagnosis and management, particularly in patients with relevant clinical presentations or family histories.

*RANBP2* is a multifunctional protein that regulates nucleocytoplasmic transport, mitochondrial function, gene expression, and immunity (Shukla et al., 2021). Specifically, *RANBP2* plays a role in regulating gene expression profiles, including for cytokine IL-6. Pathogenic *RANBP2* variants disrupt immune responses through mechanisms that are not yet fully understood, leading to marked systemic inflammation or “cytokine storms,” and contributing to systemic and CNS damage. In Case 2, elevated CSF IL-6 and IL-8 support cytokine storm involvement in ANE1 pathogenesis (Shukla et al., 2021).

Cytokines have receptors on platelets and can act as platelet activators (Bester and Pretorius, 2016). Elevated cytokines likely contribute to hypercoagulability in inflammatory conditions, including ANE, as seen in the transient thrombocytopenia in Case 1 and slightly elevated D-dimers in both cases. Disseminated intravascular coagulation in ANE exacerbates CNS tissue necrosis (Shukla et al., 2021), while systemic inflammation involving cytokines such as IL-6 promotes blood-brain barrier breakdown (Huang et al., 2021); evidenced by gadolinium enhancement and increased CSF/serum albumin ratio in Case 2. Also, there is limited evidence of intrathecal pro-inflammatory cytokine production during the ANE acute phase (Kubo et al., 2006). Given the role of cytokines in ANE1 pathogenesis, immunotherapy is the mainstay of treatment (Brattsand and Linden, 1996).

### 3.4 ANE treatment

Early, aggressive immunotherapy was essential in both cases. Case 2 received corticosteroids, PLEX, IVIG, and tocilizumab in the acute phase. Case 1 received corticosteroids, IVIG, and tocilizumab in the first ANE episode, with PLEX added in the second. Corticosteroids reduce pro-inflammatory cytokines (Brattsand and Linden, 1996) and stabilize the blood-brain barrier (Witt and Sandoval, 2014). PLEX reduces circulating inflammatory mediators and shifts proinflammatory Th1 to anti-inflammatory Th2-mediated T-cell immune responses in peripheral blood (Goto et al., 2001). IVIG may reduce cytokine-activated T-cell proliferation and suppress pro-inflammatory cytokine expression (Aktas et al., 2001). Tocilizumab, a monoclonal antibody with selective IL-6 blockade, has shown benefit in ANE1 (Preuss and Anjum, 2025; Hosie et al., 2023; Koh et al., 2019), and IL-6 may serve as a biomarker of ANE disease severity (Christou et al., 2024). Tocilizumab risks include gastrointestinal perforations, with rare reports of pneumatosis intestinalis (Vikse and Henry, 2020; Jacobs et al., 2018; Bruce-Hickman et al., 2020). Further research is needed to clarify tocilizumab’s potential benefit and optimal duration.

In the absence of standardized guidelines, long-term maintenance therapy may be considered. In the present cases, we empirically transitioned to IVIG monotherapy for long-term maintenance. Case 1 remained episode-free for over 2.5 years on IVIG. Case 2 started monthly IVIG after the most recent episode, without ANE recurrence for 2.7 years after follow-up. Bergamino et al. reported the benefit of monthly IVIG and low-dose steroid therapy in an ANE1 case (Bergamino et al., 2012). No standardized guidelines exist for ANE1 maintenance therapy, but maintenance IVIG may modulate overactive immune responses and reduce infection-triggered ANE episodes, particularly in young children at high risk of severe and fatal disease.

## 4 Limitations

This report has limitations inherent to case studies. Only patients with genetically confirmed ANE1, long-term follow-up, and consent for publication were included. Findings from two patients are not generalizable, and the absence of a control group limits causal inference. Treatments are largely informed by individual case reports, and no consensus on best practices exists. Although immunomodulator therapies have been described, comparative data are lacking, making it difficult to assess their relative effectiveness. Nonetheless, these cases contribute to the limited ANE1 literature and may inform future research.

## 5 Conclusion

These cases underscore the importance of early, aggressive immunotherapy in acute ANE1 and suggest a potential role for long-term IVIG in reducing the risk of ANE recurrence. Immunotherapy, particularly cytokine-targeting agents such as tocilizumab, holds promise in mitigating ANE’s inflammatory cascade. However, the use of tocilizumab along with the preventative role of long-term IVIG in ANE1 warrants further investigation. Neuroimaging remains central to ANE diagnosis and prognosis assessment, though defining specific features of *RANBP*2-associated ANE1 remains challenging due to limited data. Ongoing research in ANE1, including multicenter registries and databases to identify relevant biomarkers and systematically assess clinical responses to therapies, is essential to improve understanding, treatment, and outcomes in this rare but severe condition.

## Data Availability

The original contributions presented in the study are included in the article/Supplementary Material, further inquiries can be directed to the corresponding author.
