# Supplementary material for: Case Report: long-term clinical outcomes in RANBP2-associated acute necrotizing encephalopathy
Source: Front Pharmacol. 2025 Jun 5;16:1607682. doi: 10.3389/fphar.2025.1607682 (PMC12176811; doi:10.3389/fphar.2025.1607682)
Supplement: Supplementary file 1 [file DataSheet1.pdf]

Supplementary Material

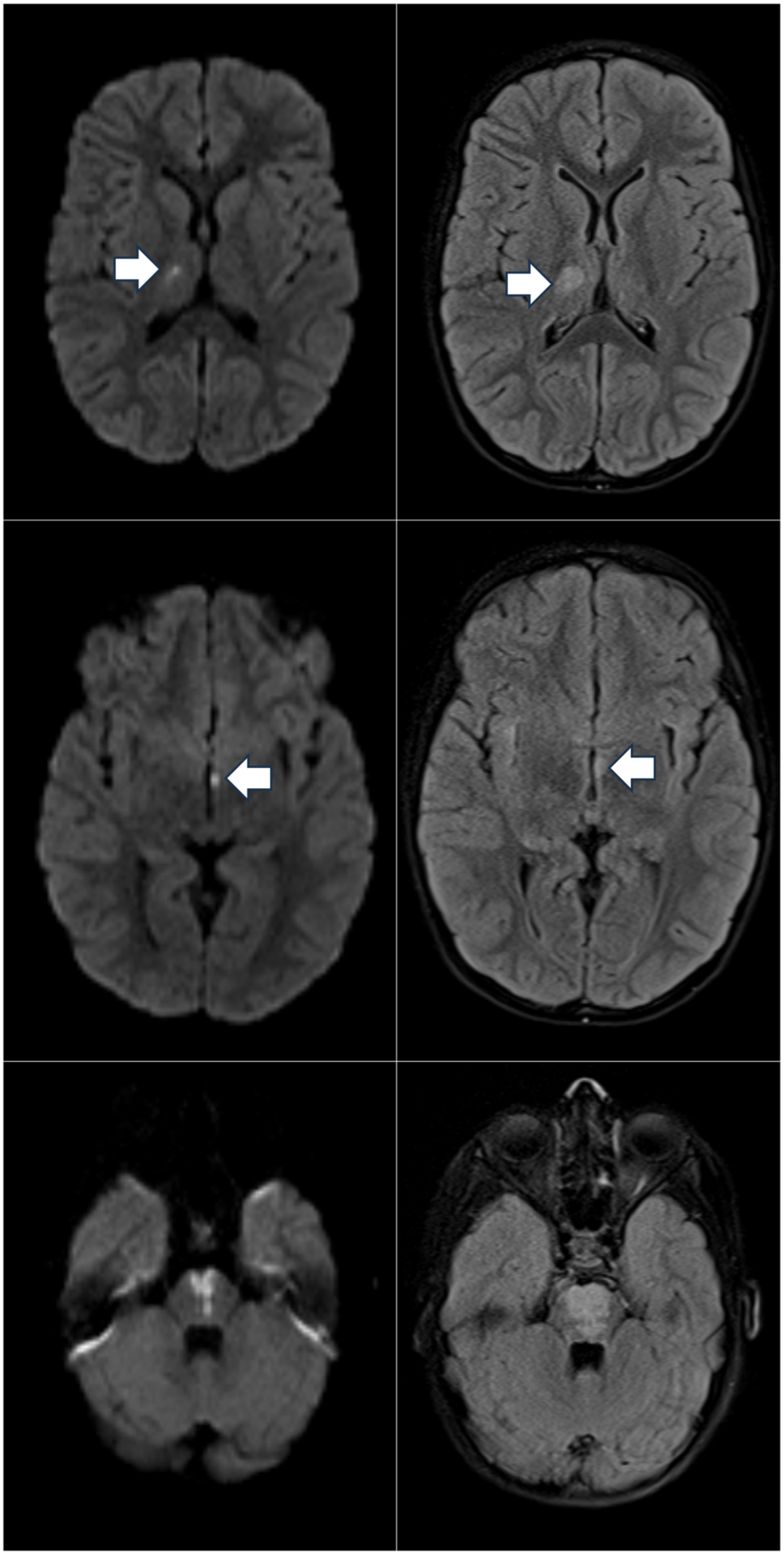

Supplementary Figure 1: Case 1 MRI. Case 1 brain MRI at age 4 years, 10 months shows foci of restricted diffusion and hyperintensities in bilateral thalami (arrows) and brainstem on the diffusion-weighted imaging (left column) and FLAIR images (right column).

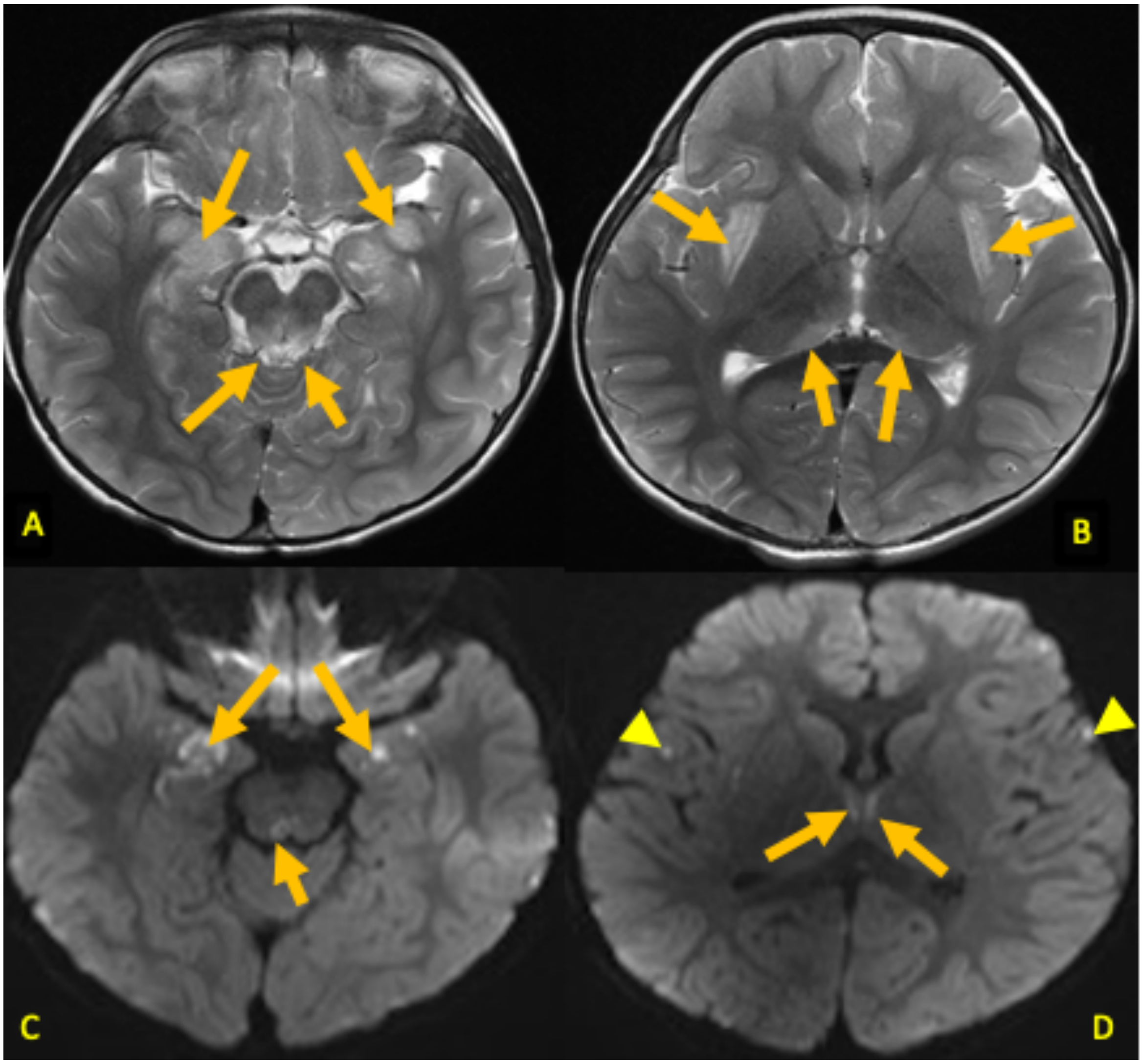

Supplementary Figure 2: Case 2 MRI at age 30 months. Case 2 brain MRI obtained during the second hospital admission shows axial T2-weighted images (A, B) demonstrating patchy hyperintense signal abnormalities, and axial diffusion-weighted images (C, D) demonstrating associated diffusion restriction (ADC not shown) involving medial temporal lobes, periaqueductal area, medial thalami, consistent with acute necrosis. Additional punctate foci of diffusion restriction are also noted in frontal cortices, likely due to postictal changes. {Adapted from Olubiyi et al., BJR Case Rep 2022}

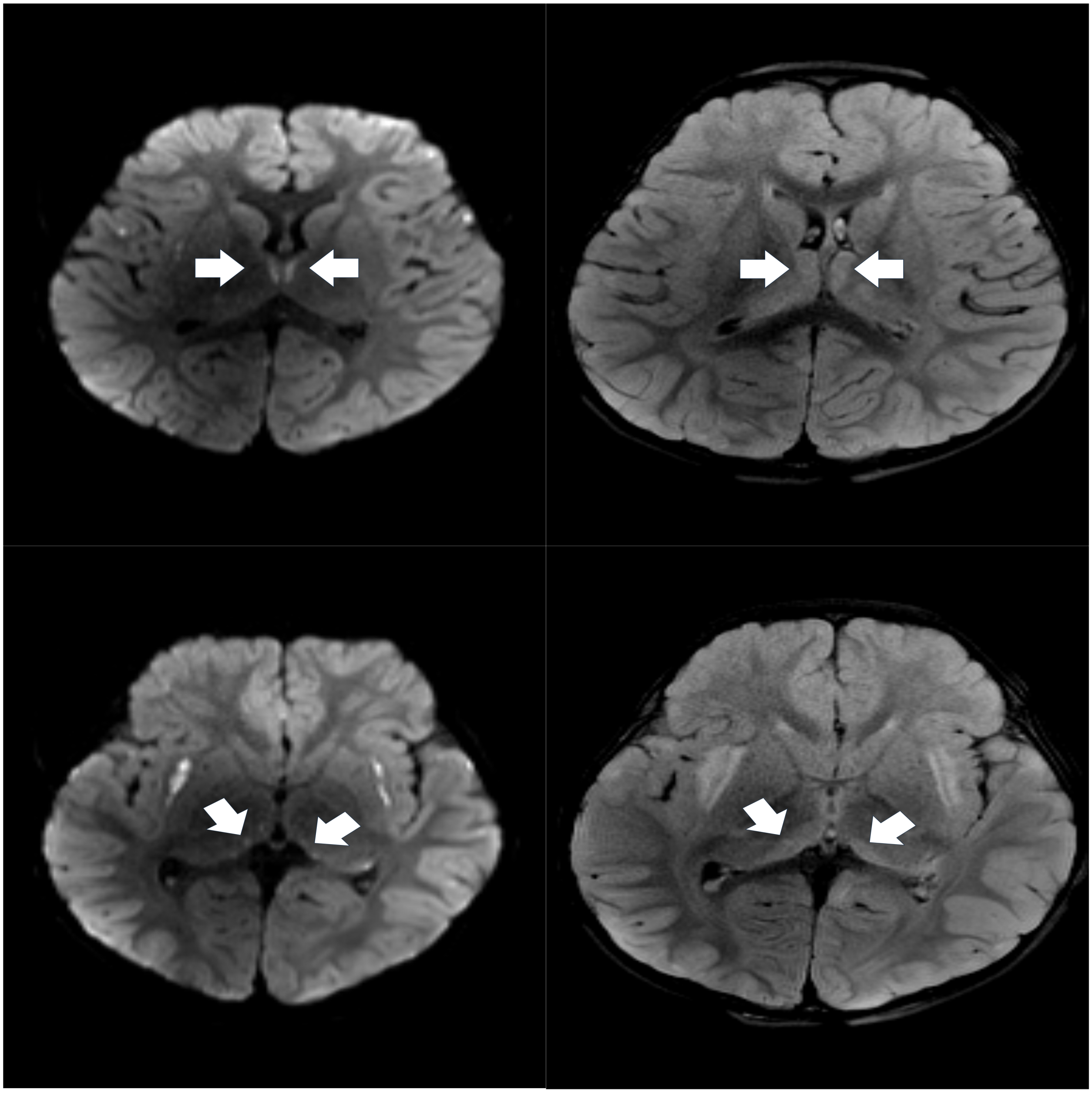

Supplementary Figure 3: Case 2 MRI at age 40 months. Case 2 brain MRI during the third admission at age 40 months shows foci of restricted diffusion and FLAIR hyperintensities in bilateral thalami (arrows) and brainstem on the diffusion-weighted imaging (left column) and FLAIR imaging (right column).
